# A qualitative evaluation of a brief multicomponent intervention provided by lay health workers for women affected by adversity in urban Kenya

**DOI:** 10.1017/gmh.2017.26

**Published:** 2018-02-06

**Authors:** Edith van't Hof, Katie S. Dawson, Alison Schafer, Anna Chiumento, Melissa Harper Shehadeh, Marit Sijbrandij, Richard A. Bryant, Dorothy Anjuri, Phiona Koyiet, Lincoln Ndogoni, Jeannette Ulate, Mark van Ommeren

**Affiliations:** 1 Department of Mental Health and Substance Abuse, World Health Organisation, Geneva, Switzerland; 2 University of New South Wales, Sydney, Australia; 3 World Vision International, Burwood East, Victoria, Australia; 4 University of Liverpool, Liverpool, UK; 5 VU University, Amsterdam, Netherlands; 6 World Vision Kenya, Nairobi, Kenya; 7 Psychosocial Support Center, Nairobi, Kenya; 8 World Vision Canada, Missossauga, Canada

**Keywords:** Adversity, intervention, mental health

## Abstract

**Background:**

Problem Management Plus (PM+) is a brief multicomponent intervention incorporating behavioral strategies delivered by lay health workers. The effectiveness of PM+ has been evaluated in randomized controlled trials in Kenya and Pakistan. When developing interventions for large-scale implementation it is considered essential to evaluate their feasibility and acceptability in addition to their efficacy. This paper discusses a qualitative evaluation of PM+ for women affected by adversity in Kenya.

**Methods:**

Qualitative interviews were conducted with 27 key informants from peri-urban Nairobi, Kenya, where PM+ was tested. Interview participants included six women who completed PM+, six community health volunteers (CHVs) who delivered the intervention, seven people with local decision making power, and eight project staff involved in the PM+ trial.

**Results:**

Key informants generally noted positive experiences with PM+. Participants and CHVs reported the positive impact PM+ had made on their lives. Nonetheless, potential structural and psychological barriers to scale up were identified. The sustainability of CHVs as unsalaried, volunteer providers was mentioned by most interviewees as the main barrier to scaling up the intervention.

**Conclusions:**

The findings across diverse stakeholders show that PM+ is largely acceptable in this Kenyan setting. The results indicated that when further implemented, PM+ could be of great value to people in communities exposed to adversities such as interpersonal violence and chronic poverty. Barriers to large-scale implementation were identified, of which the sustainability of the non-specialist health workforce was the most important one.

## Introduction

Exposure to adversities such as interpersonal violence, chronic poverty, long-term armed conflict, and displacement are risk factors for common mental health problems, including depression, anxiety disorders, and posttraumatic stress disorder (PTSD). An estimated 35% of women worldwide report having experienced physical and/or sexual violence (World Health Organization [WHO], 2013). Women exposed to adversity and violence have a higher risk of developing common mental health problems (Heise and Kotsadam, 2015). The human, financial and other health systems resources in low and middle-income countries (LMIC) are often too scarce to scale-up mental health care, and often mental health problems associated with adversity go untreated (Jacob *et al.* 2007; Prince *et al.* 2007; Saxena *et al.* 2007).

Mental health interventions that are brief, deliverable by non-specialist health providers and address multiple outcomes are more likely sustainable and potentially scalable in settings with limited mental health resources. WHO has begun to develop and release scalable psychological interventions as part of its mental health Gap Action Programme (mhGAP) (WHO, 2008). This now includes the manualized intervention Problem Management Plus (PM+), developed by WHO and the University of New South Wales. PM+ is a brief, multicomponent behavioral intervention that can be delivered by non-specialist health providers as well as mental health specialists (Dawson *et al.* 2015; WHO, 2016). It comprises five individual face-to-face sessions (90 minutes duration) that aim to support a person's capacity to manage their own emotional distress by way of behavioral activation, stress management techniques to reduce physiological arousal, and improve one's social support and problem-solving abilities. Techniques are rehearsed in session and participants are expected to practice them between sessions. These techniques are evidence-based with proven efficacy in LMICs and recommended in WHO guidelines for common mental health problems (Dua *et al.* 2011; Tol *et al.* 2013). PM+ also provides education about common reactions to adversity and the final session addresses relapse prevention.

To gather evidence on the efficacy of PM+, a randomized controlled trial (RCT) was conducted in Kenya (Bryant *et al.* 2017) and Pakistan (Rahman *et al.* 2016), with both studies showing PM+ to be effective in reducing common mental health problems compared with a treatment as usual group. Other studies have also shown the effectiveness of task-shifting approaches, employing local non-specialist health provider (Ertl *et al*. 2011; Bass *et al*. 2013; Chibanda *et al.* 2017; Patel *et al.* 2017).

This paper discusses findings from qualitative interviews gathered from stakeholders involved in an RCT to evaluate PM+ in Kenya. In Kenya, PM+ was evaluated in peri-urban areas in Nairobi with 518 women exposed to adversity, including interpersonal violence (Dawson *et al.* 2016; Sijbrandij *et al.* 2016; Bryant *et al.* 2017). The Kenyan Health Survey in 2014 found that 54% of Kenyan women between 15 and 49 years reported having experienced physical violence, and 20% sexual violence (Kenya National Bureau of Statistics *et al.* 2015). Adverse living conditions, including but not limited to chronic poverty and living in slums, are identified as risk factors for common mental health problems (UN-Habitat, 2013). The results of this RCT showed that over 85% of women enrolled in PM+ attended all five sessions. In addition, women experienced a reduction of psychological distress, posttraumatic stress, and functional impairment 3 months after receiving PM+ (Bryant *et al.* 2017).

The impact of a psychological intervention is not only dependent upon its efficacy, but also on its uptake (i.e. a number of individuals and organizations, which use the intervention). The uptake of a psychological intervention is dependent on it being successful in reaching people who need it, organizations integrating the intervention into their services and the sustainability of the intervention (Glasgow *et al.* 1999). Optimizing the chances of these processes being conducted requires an understanding of the intervention's acceptability and feasibility for continued delivery in a specific setting. The literature identifies potential barriers to scale-up and sustainability of evidence-based psychological interventions, including a lack of human resources trained in mental health, attrition of trained non-specialist health providers, lack of mental health leadership in public health, difficulties integrating the intervention into primary care, policy and logistical challenges, insufficient funding and stigma (Saraceno *et al.* 2007; Nkonki *et al.* 2011; Padmanathan and De Silva, 2013; Murray *et al.* 2014). Evaluating the feasibility of continued delivery of interventions in a specific setting (translation of evidence to practice) is necessary to ensure uptake and implementation, as well as improve integration of interventions into routine care (Tansella and Thornicroft, 2009; Thornicroft *et al.* 2011; Peters *et al.* 2013). Information about the barriers and facilitators to intervention implementation may also be used to inform future efforts to sustainably scale up psychological interventions in other countries.

Accordingly, this paper examines the acceptability of PM+ and possible barriers and facilitators of implementing PM+ as perceived by different stakeholders involved in an RCT in Kenya. The acceptability will be explored by evaluating engagement and stakeholders’ experiences with PM+. The exploration of barriers and facilitators of implementing PM+ will give an idea of the feasibility to scale up PM+ outside the research setting and integrate it in routine service provision.

## Methods

### PM+ project in Kenya

A qualitative process evaluation was conducted in the final phase of a large research study, and was preceded by an RCT evaluating the efficacy of the PM+ intervention in Kenya. PM+ was evaluated among women affected by violence in World Vision Kenya's peri-urban Riruta Area Development Program (ADP), Dagoretti sub-county in Nairobi City County. In this region women impacted by violence usually receive minimal or no formal mental health support. As in many LMICs, there is a scarcity of specialist mental health workers. Therefore, to increase the reach of mental health care for women PM+ uses a task-shifting approach where the delivery of healthcare is ‘shifted’ from a specialist (e.g. psychologist or psychiatrist) to other cadres of health workers (e.g. nurses) or non-specialist health providers. The RCT compared PM+ with enhanced usual care in 518 women exposed to adversity, approximately 81% of whom had a history of gender-based violence (GBV). The inclusion criteria for participation were psychological distress (measured by the General Health Questionnaire-12) and impaired functioning (measured by WHO Disability Assessment Schedule). The PM+ manual was translated and contextually adapted by local mental health experts and CHVs to ensure the appropriateness and acceptability of PM+ to the local setting.

CHVs were selected by Kenya's Ministry of Health (MoH) and interviewed for suitability to be trained as PM+ providers. CHVs were considered as ideal providers as they were already conducting health-related activities within the communities where the RCT was taking place and were a source of support for women. In their existing role, CHVs had received government training in basic health care, but did not have previous training or experience in mental health care. CHVs received 8-days classroom training by the PM+ Master trainer (KSD) and completed supervised PM+ practice cases before delivering the intervention. All CHVs were assessed for their competency in delivering PM+ before offering it to RCT participants. PM+ participants received 5 weekly individual PM+ sessions. CHVs received monetary compensation for their role as PM+ providers. Two local experienced psychologists were trained as PM+ supervisors and supervised CHVs on a weekly basis during the RCT. Supervisors were also supervised by the aforementioned Master trainer (KSD). Fidelity checks were conducted to ensure the intervention was delivered as per the manual. Previous publications describe further information on the study (Dawson *et al.* 2016; Sijbrandij *et al.* 2016; Bryant *et al.* 2017).

### Data collection

In-depth interviews (IDIs) were conducted with 27 key informants. These were: (a) six PM+ participants from the three areas in which the project was implemented; (b) six CHVs (PM+ providers); (c) seven decision makers (two community chiefs, the operations director at World Vision Kenya (WVK), the head of quality and assessment in the WVK office, a chief nurse at a participating health care facility, a district public health nurse and the MoH sub-county head); (d) seven project staff (three assessors, two PM+ clinical supervisors, one principal investigator, and one independent trial monitor); and (e) one Community Health Extension Worker (assigned in Kenya's primary health care clinics to coordinate and provide daily management of clinical activities and CHVs). Due to resource limitations, participants from the control condition or those who refused participation or dropped out from PM+ were not included in this qualitative study, which may have caused bias in the data. Participants for this qualitative study were randomly selected and data were collected by an interviewer (EvtH) not involved in the RCT. The IDIs took place in locations convenient to key informants (e.g. participants’ homes, WVK office, Community Health Center) and followed a semi-structured guide comprising a series of open-ended questions (Appendix A) about the PM+ program, including: barriers and facilitating factors to treatment engagement and adherence; barriers and facilitating factors for large-scale implementation of PM+; and perceptions of the benefits and challenges of integrating PM+ into CHVs routine service provision. IDIs were facilitated by an independent interviewer who had not been involved in the project, and were conducted in English or in the local language, with an interpreter fluent in English and the main local languages spoken in the region (Kiswahili and Kikuyu). Each IDI lasted between 30 and 60 min. Interviews were not audio recorded due to participants’ concerns about privacy and safety. Notes were made by the interviewer and interpreter simultaneously during the interviews and were later compared for inconsistencies that were discussed and if necessary checked with the participants to ensure clarity.

### Ethics

Written informed consent was obtained from all participants, including consent to the reporting of research results. The project was approved locally by the Research Ethics Committee of the Great Lakes University of Kisumu, Kenya and by the WHO Ethical Review Committee (Protocol ID: RPC656, April 25, 2014, Amendment February 16, 2015), with approvals including this qualitative process evaluation of the overall research initiative.

### Analysis

Qualitative data were analyzed thematically by one of the authors (EvtH), following the framework approach (Pope *et al.* 2000). After initial familiarization of the data, a thematic framework that comprised main themes and subtopics was developed. Thereafter all data were indexed and coded according to this framework, with charts of themes made for mapping and interpretation of the data. The participant subgroups were analyzed individually, before triangulating the data across groups to identify common themes. The interviews were explored deductively in relation to the research questions whilst also allowing inductive analysis for new emerging themes. All interview transcripts were coded in NVIVO11 (QRS International, 2015).

## Results

The results of the qualitative interview are presented by key themes and incorporate quotes and findings from across all stakeholder groups. Central aspects such as the sustainability of non-specialist health providers, integration of primary health care (PHC) and training and supervision are further explored in the discussion.

### Experiences of PM+

All the interviewed participants and CHVs shared positive experiences of the PM+ intervention. Participants reported positive changes PM+ had made in their lives. One PM+ participant reported: *‘I was in a really bad state before the program, had no interaction with people, hated noise, got irritated and angry easily. Even my own children were too much…. now I can enjoy them and my life and help others’.* One CHV mentioned: ‘*The strengthening social support skills helped women a lot. One woman was struggling to get her child into school (due to school fees, uniform, no money for bus fare) and when opening up to others about her problems she got connected to people that could help her and now her child is going to school*’. The CHVs identified positive changes PM+ had made for them personally, such as feeling more knowledgeable about the effects of stress and better equipped to serve their community, managing their own problems and noticing improved general well-being in themselves. Table 1 shows the ‘changes’ resulting from participation in the PM+ program mentioned by both CHVs and PM+ participants, which included mental, behavioral, interpersonal, physical and knowledge changes.

**Table 1.** Reported changes in PM+ participants as mentioned by CHVs and PM+ participants

Domain	Example of changes mentioned
Mental	Being able to enjoy things more, feeling better about oneself, feeling empowered
Behavioral	Increase in general activities, improved self-care and being able to do household chores
Interpersonal	Engaging more with other people, having fewer arguments, seeking support
Physical	Improved sleep, fewer headaches or lower blood pressure
Knowledge	More knowledge on how to respond to stress and problems (e.g. tools to better deal with problems, strategies to reduce worry and stress)

Participants and CHVs were clear about the potential value of scaling up PM+ and the positive effect it could have on the community. CHVs and four PM+ participants indicated they were approached by people that had heard about PM+ and wanted to participate, suggesting PM+ was perceived positively by the community.

### Acceptability and feasibility of PM+ content

PM+ participants mentioned they found the four strategies (stress management, behavioral activation, problem-solving and strengthening social support) very useful. One participant reported that only the stress management strategy was new for her and that she had previously been using the other strategies. CHVs and participants reported that the stress management strategy was applied by participants more than other PM+ strategies, reporting that for most participants this strategy was easy to understand and practice because of the clear explanation provided by PM+ providers. It was reportedly helpful for participants to rehearse the strategies in session and learn how they could be applied to their lives through home practice. Some participants mentioned they would have preferred more practice time with the CHV, for example by increasing the number of sessions, including a refresher session following completion of PM+, or creating peer groups to practice the strategies together.

The problem-solving strategy was reported by CHVs as sometimes being difficult to explain to participants. It also was identified as the PM+ strategy that may be the most challenging to implement as some participants chose problems that were likely unsolvable. As one CHV reported: ‘*They (PM+ participants) use the skills (of problem management), but have no money to start something* [*e.g., a business*], *so still get stuck. They have the skills and knowledge now, but no finances to steer their ideas*’. Another CHV mentioned: ‘*(the) implementation gets stuck because client does not have money or goods to start business*’.

### Relationship between CHVs and participants

The interviewed participants and CHVs identified positive therapeutic relationships. One participant mentioned: ‘*(my) contact with the CHV was very good. She was calm and warm and approachable. Which made it easy to open up*’. A CHV reported: ‘*I had very good relationships with all PM+ participants*.’

Participants and CHVs often mentioned that initially, it was very difficult for participants to open up. However, CHV assurances of confidentiality of information shared in sessions likely encouraged PM+ participants to open up, as described by a participant: ‘*Revealing personal information in the beginning was difficult, but because confidentiality was assured I was able to open up*’. Some CHVs shared that the development of a therapeutic relationship was fostered when the CHV and the participant did not already know each other and where confidentiality was assured: ‘*It works better if the CHV does not know the client. People are often scared to tell their friends about problems because of gossip. If you tell a friend about a problem, then soon everybody knows*’.

### Perception of participants’ families

All the participants had informed family, friends or neighbors about the intervention and reported that they were all supportive of the participants’ involvement. PM+ providers also engaged and informed family members of PM+ participants about the intervention to demystify the program and improve participant attendance. In these instances, CHVs reported good experiences with families of the participants. Positive experiences with husbands of participants were mentioned by all interviewed CHVs. This included husbands accepting the intervention, giving their wives privacy to conduct the sessions, collaborating with CHVs to help their wives to participate, lending their cell phone for communication with CHVs, and showing appreciation towards the CHVs for the positive changes they saw in their wives. The changes mentioned by husbands to CHVs were usually that they could communicate better, were arguing less, and that their wives were taking better care of themselves and their household or family. Conversely, two CHVs mentioned negative experiences with participants’ husbands: ‘*Some (husbands) were negative about the program and did not allow their wives to be in the program. If a husband was there (at house) I would sometimes pretend it was on normal hospital visit. Some(PM+) participants would not open the door if the husband was there, some would make arrangements to meet somewhere else.*’

### Barriers and enabling factors to engagement of PM+ participants

Key informants identified structural, attitudinal, and psychological barriers and facilitating factors in adhering to the intervention; and using the skills taught. Each is discussed below.

The main structural barrier to organizing sessions mentioned by participants and CHVs was that many participants and CHVs would accept casual labor. This made it difficult to plan sessions around last-minute work opportunities. Women could not afford to stay at home to wait for CHVs if they had been offered work, and CHVs sometimes missed out on daily work because participants canceled sessions at the last minute. Not being dependent on other people for jobs, for example working in their own gardens, was mentioned by CHVs as a facilitating factor, as these women were more flexible with their work schedules. Having pre-scheduled appointments was identified as the best way to ensure people attended sessions regularly. Good communication was mentioned as being beneficial to making appointments mutually convenient to CHVs and participants. Another perceived structural barrier mentioned by CHVs was that some participants dropped out after the third session because their symptoms improved. Despite this, CHVs reported that most participants were committed and tried to complete all the sessions.

Most participants reported that delivering the intervention sessions in their home was an advantage, as one participant mentioned ‘*I could finish all the 5 sessions because it was easy to plan them. I did not have to go anywhere*’. Conversely, some CHVs identified home-delivery disadvantageous because of the perceived potential for shaming and stigmatization of participants if neighbors observed the CHVs regularly attending the same household. One CHV mentioned: ‘*It would be easier for the women to go to a specific centre instead of the treatment provider coming to them, because of stigma in the society. A centre will provide more privacy as compared to clients*’ *homes. For example sometimes husband would come in etc.*’

The main attitudinal barrier mentioned by CHVs was participants’ expectations of monetary support. CHVs mentioned that despite making it very clear that financial support was not part of the PM+ program, many participants still expected such support, for example, to start a business or school fees. As one CHV mentioned: ‘*Perceptions of people (*participants*) were a challenge. The program (*referring to World Vision's substantial development support in the area*) has money and they had different expectations than what the program (PM+) could give’.* Another CHV mentioned: ‘*Some (PM+ participants) were in a very bad state and expected material help which the program could not provide, and dropped out’.*

Other barriers mentioned by CHVs included participants not being accustomed to self-identifying solutions to their problems (because usually people would tell them what to do), not believing the intervention could help them, challenging living circumstances of participants that evidently could not be improved (e.g. financial problems or caring for disabled dependents), and pride that meant they would not accept help.

CHVs suggested improving participant engagement via PM+ group meetings, shortening the length of sessions (currently 90 min) or giving tangible contributions (e.g. a token in the form of money or a small bag with the project logo) to participants after every session to motivate continued engagement. It was suggested that providing financial support in starting a business would be an effective way to engage participants in the intervention. After completing the intervention having a follow-up or booster sessions, or forming peer groups were mentioned as ways to encourage participants to keep using the strategies. Improved understanding of one's own problems and seeing the benefits of the intervention were considered to increase participants’ engagement in PM+ according to CHVs. However, this qualitative evaluation suggests that even with these features, interview respondents still request for material benefits. This suggests that poverty is a key factor affecting successful implementation of programs.

Psychological barriers to intervention engagement were mentioned by CHVs and participants. CHVs often spoke about participants disclosing they felt ashamed opening up about their problems and believed they were to blame for their own problems, something that was reinforced by interviews with participants. Distrust of strangers such as being approached by World Vision staff who introduced the PM+ project was mentioned as a barrier to recruiting people.

Lack of mental health awareness and stigma were identified by decision-makers and project staff as barriers to scale up. Interviewees explained the existence of community stigma towards mental health problems and a lack of mental health awareness. Thus even when people do recognize their symptoms they may not seek help or seek help late.

Mental health promotion, education, and other awareness activities were suggested by decision makers as ways to address community stigma towards mental health, whilst also making PM+ more acceptable. It was suggested these activities involve key community members, such as chiefs, religious and traditional healers, and police. Another suggestion to minimize stigma was to integrate PM+ into other services, for example into income-generating activities, so that other community members will not know people are receiving help for mental health problems.

Decision makers and project staff identified that wider mental health awareness and prioritization of mental health is essential for the intervention to be scaled up successfully. It was mentioned by some that current mental health is not prioritized enough by decision makers, despite likely being an important factor to stimulate development in communities. National level projects tend to prioritize monetary and other more direct poverty relief programs, making it difficult to obtain support for mental health projects.

### Integration and scale-up of PM+

All decision makers mentioned that the integration of PM+ into the PHC system would be feasible. The structure of the PM+ intervention is in line with the MOH strategy to use community-based approaches to healthcare delivery (as described in the Kenya Mental Health Policy 2012-2030). The PHC in Kenya offers direct links to communities, making it possible to reach a large number of people. CHVs were perceived as good delivery agents and are directly connected and operating within Kenya's PHC system making delivery in this system a sensible approach.

Lack of funding for human resources required to deliver PM+ in PHCs was seen as a possible key barrier to integration, as well as addressing stigma and discrimination towards mental health to promote mental health help-seeking behaviors. One local decision maker mentioned: ‘A *barrier is fact that CHVs are volunteers and not on government payroll. They were supposed to be, but there is not enough budget*’. Advocacy at the government level, such as sharing the results and recommendations from the PM+ study at national level forums, was identified as crucial to ensure government strategies and budgets prioritize mental health interventions.

Integration of PM+ into services other than those that are health-related was suggested by interviewees: ‘*Mental health should be integrated in other services. If you don't integrate then there will be a lot of stigma.*’ It was suggested by decision makers that PM+ implementation could also be conducted through chiefs’ offices since a lot of people approach chiefs with their problems. Another suggestion was integrating PM+ into development programs to better engage participants. Finally, adding practical income-generating strategies could ensure participants can meet their own basic needs and adhere fully to the intervention, as could the addition of activity programs or PM+ support groups. *Chamas* (women groups) were mentioned as another possible place to integrate PM+ to increase coverage, and as a possible way to support opening up and sharing among women.

### Facilitators and barriers to using CHVs as PM+ providers

Among all decision makers, CHVs were seen as the most suitable people to provide the intervention. Because of their relationship with elders, the CHVs are in a position to identify individuals in the community who have psychological needs. Although participant's mentioning concern about confidentiality they also felt CHVs made appropriate delivery agents as many people in the community know them and may trust them, making it easier for people to open up about their problems. Another facilitating factor mentioned in working with CHVs is that they all have had government training in basic health care.

Project staff and decision makers indicated that the selection of CHVs as delivery agents of PM+ should be more thorough to ensure they are competent to undertake the PM+ provider role. Desirable selection criteria suggested by decision makers are integrity, eagerness to learn, teachable, good communication skills, and some background in counseling. Project staff added the criteria of older age, a passion to serve the community, good interpersonal skills, a basic level of education, acceptance by the community, and fluency in the local language. It was mentioned that CHVs often have the same issues as participants and that CHVs who experience psychological problems should first complete the intervention as a participant, enabling them to become a role model too.

Another barrier mentioned was the provision of sufficient supervision and training of CHVs. Most project staff and decision makers indicated that the amount of supervision provided to CHVs for their role as PM+ provider was insufficient, suggesting intensifying the supervision by increasing supervision time and creating opportunities to share their experiences as PM+ providers. Project staff claimed that some CHVs were experiencing stress associated with working with participants with common mental health problems (e.g. not sleeping well, worrying). One CHV mentioned: ‘*I would feel traumatized by some cases and would take it back home and have nightmares and deal with it myself. The supervision was not enough; one-on-one sessions with supervisor to talk about our own experiences would be nice.’*

An identified barrier to supervision was the amount of time and budget required for supervision. CHVs suggested it would help them to have CHV-peer groups to share their experiences with one another, which could partly address this barrier.

Regarding the classroom training, some decision-makers and project staff felt that it could be improved by making it more extensive. They found the training too brief, covering too much information in a short amount of time. They suggested extending experiential learning and simulation activities as part of training. There was a perceived gap between the trainer and CHVs in background and experience in the local context. It was suggested that more dialogue between foreign and local professionals is needed to tailor the PM+ content and delivery methods to match the socio-cultural norms in the target community. Other suggestions were to provide more training on recognizing mental illnesses, having refresher trainings, and providing more individualized support for the CHVs on issues that are relevant to them.

Though the CHVs received financial incentives for their PM+ work, the sustainability of CHVs as unsalaried, volunteer providers was mentioned as the biggest potential barrier to scaling up the intervention by CHVs, project staff and decision makers. Project staff suggested that keeping CHVs engaged as volunteers would be problematic because the work is too intensive to be considered a volunteer job. They reported that some CHVs became demotivated due to not receiving adequate remuneration throughout the research project and having to find time for income-generating work or household chores, a factor also mentioned by CHVs. Project staff suggested CHVs should be contracted by health care facilities or to find another way to provide CHVs with more financial support so that they can combine managing their personal lives and serving the community. Decision makers also mentioned the provision of a monthly, stable income to improve CHVs dedication to the job. Another suggestion was to influence CHVs to appreciate other forms of motivation besides money by enhancing their skills, thereby increasing intrinsic motivation. Suggestions for better integration of CHVs within the health system are to give them more financial compensation for their work on PM+. Advocacy to prioritize mental health more that could lead to policy changes was also mentioned as beneficial to integration.

The use of nurses as PM+ providers was seen by decision makers as an alternative option to CHVs because they are already on the payroll of the health care system and have basic mental health training, but the lack of personnel in these roles and their overwhelming workload were identified as barriers to this idea. If nurses were to provide the intervention interviewees felt the nursing workforce should be expanded, should receive more training on recognizing and addressing mental health difficulties, and should have time allocated to provide this intervention as part of their routine role.

### Other suggestions for improvement

To improve coverage of treatment for mental health problems all key informants suggested the intervention should also be offered to men and adolescents. One participant mentioned: ‘*Involve men in the program. This will help them manage their own stress. They don't know how to handle stress and use violence to express stress*’, something echoed by a CHV who reported: ‘*involve men in the project, it is not easy for women to manage stress of the husband*’.

## Discussion

The findings show that PM+ was largely acceptable to all key informants involved in the project, but barriers to scale up were identified. The main barriers identified were the sustainability of CHVs as PM+ providers in routine service. All key informants indicated that PM+ could be valuable for individuals and communities exposed to adversities. Participants and CHVs reported emotional, behavioral, interpersonal, and physical improvements of participants and a general positive impact the intervention had made on their lives. This is consistent with the positive results from the RCT conducted in Pakistan (Rahman *et al.* 2016) and Kenya (Bryant *et al.* 2017).

PM+ was largely seen as being viable for integration into the PHC system using the existing CHV staffing structure. However, the sustainability of this approach faces challenges in selection, training, supervision, and compensation of the CHVs. So, although the evidence supporting the effectiveness of non-specialist health providers with no prior specific training to deliver complex interventions is growing (van Ginneken *et al.* 2013; Chibanda *et al.* 2016) challenges are identified in the sustainability of this approach. The challenges uncovered by this study and others (Glenton *et al.* 2013; Chowdhary *et al.* 2014; Abas *et al.* 2016) in the selection, training and supervision of staff that deliver the intervention need to be addressed as they are important for the sustainability of a task shifting approach (Mendenhall *et al.* 2014). Even though cadres like CHVs can effectively deliver interventions like PM+, the main challenge is creating a sustainable system around them that will allow them to perform well. Training, supervision and monetary compensation are likely needed for such a system. To implement programs like PM+ at scale, it is essential to develop recruitment protocols, deliver adequate training, to provide high-intensity supervision and quality assessment structures that are sustainable, which requires a sustainable financing system.

Health worker motivation is crucial for successful implementation of an intervention (Kok *et al.* 2015). This study showed that, overall, CHVs were not satisfied with the monetary compensation received during the project for being a PM+ provider and wanted more supervision offered for this emotionally demanding role. Non-specialist providers in other studies evaluating psychological interventions have reported similar levels of job dissatisfaction, notably citing low motivation and increased work pressure (Chowdhary *et al.* 2014; Abas *et al.* 2016). This evaluation adds to the literature on challenges to implementing task-shifting approaches in mental health (Padmanathan and De Silva, 2013; Mendenhall *et al.* 2014). During our trial, CHVs were compensated for their role as PM+ providers because there was funding for the study. Scaling up PM+ in routine health systems would unlikely involve compensation for additional workloads, and would thus potentially generate a much heavier burden on an unpaid workforce. The lack of government resources (financial and personnel) allocated to mental health services in LMIC is a barrier to implementing psychological interventions and risks causing the services to be short-term and reliant upon outside grant funding. To make task-shifting interventions scalable, urgent action to explore models of sustainable financing, including remuneration of providers, is required (Murray *et al.* 2014). Recent return on investment research could provide compelling evidence of the potential gains from investing in mental health care (Chisholm *et al.* 2016).

As in other studies (Chowdhary *et al.* 2014; Abas *et al.* 2016), CHVs were able to deliver PM+ and develop a therapeutic relationship with the participants. However, whether they are the ideal providers of mental health interventions was not agreed upon. The opinions about CHVs as PM+ providers were mixed with some participants finding it easy to trust them while others did not. In this project, it seems that the initial contact and engagement in the program is easier when participants are approached by someone they do know. However, the sharing of personal information and problems was possibly easier when participants do not have an established relationship with the non-specialist providers. As reported in other studies (Chowdhary *et al.* 2014), ensuring confidentiality is important in participants opening up about their problems. It seemed that although CHVs were community-based, they did have to work to gain people's trust to open up. More research should be conducted on the role of trust within these processes to inform future program development, training, and case identification.

Lack of mental health awareness and stigma were mentioned as barriers that need addressing at multiple levels: individual, community, and policy. Mental health awareness and other educational activities in collaboration with key community figures were suggested as strategies to increase the acceptability of PM+ and mobilize the community to participate in the intervention. Making mental health a priority in decision making and funding organizations will be necessary to create the support needed to scale up PM+. Shame and guilt about their problems (including being victims of violence) were identified by women as possible barriers to seeking help and should be addressed to increase a help-seeking behavior (Hegarty *et al.* 2016). The integration of GBV awareness activities and care for GBV victims into existing health services will contribute to reducing stigma and will make it easier for women to access health services (IASC, 2015).

In contrast to the RCT in Pakistan (Rahman *et al.* 2016), men were not included in this RCT in Kenya because the focus of the research was to investigate the effect of PM+ on women affected by GBV. A recommendation to improve the program's reach was to include men and adolescents. The need to include men, as they are too often perpetrators of violence against women, is important to consider in further implementing PM+. Including boys and men has shown promise in a program aimed at preventing GBV (Ricardo *et al.* 2011; Hossain *et al.* 2014). Importantly, the PM+ study in Pakistan found the equal effectiveness of the intervention for men and women exposed to adversity, suggesting that exploring a universal approach delivering to both men and women in Kenya could be beneficial (Rahman *et al.* 2016). In Kenya, a group format for men is being considered with an aim to reduce harmful alcohol use and ultimately, intimate partner violence (Schafer and Koyiet, 2017). Many common mental health problems emerge in adolescence, rendering it a vulnerable time, and therefore investigating the effectiveness of PM+ for adolescents is also warranted.

A key implementation challenge was overcoming mismatched expectations about what the PM+ program provided. Participants received repeated explanations about the aim of PM+ and that financial assistance was not part of the program. They received this message at the screening and informed consent phases of the trial, as well as by the PM+ providers during sessions. Despite this, participants’ expectations of tangible and financial assistance from PM+ persisted. A possible explanation for this can be found in the history the population has with international organizations within their community, including familiarity with World Vision as an organization that has previously offered financial and practical program initiatives.

When providing psychosocial assistance to low-income communities who are used to financial assistance, a challenge is to convince people that non-financial assistance may be helpful to them as well. One possible way to minimize the risk of a mismatch in expectations would be to scale-up through systems in which financial assistance is not expected, such as the government health system. Actively managing community expectations around PM+ by conducting anti-stigma and mental health and PM+ awareness activities could be beneficial. Creating programs that link mental health and economic activity could be another way to increase the feasibility of providing psychosocial support in poor communities. Creating group formats of the intervention was mentioned by respondents as a possible way to increase motivation by providing a forum for women to help one another by sharing livelihood opportunities and experiences. The potential additional benefits of a group version of PM+ – including the role of peer support and sharing of experiences – are being actively explored in Pakistan (Chiumento *et al.* 2017), Nepal, and in Kenya with men (Schafer and Koyiet, 2017). This study calls for more research on the way mental health programs are presented to the community and how engagement can be increased.

Another challenge related to poverty and financial assistance was that financial problems were often chosen as the focus of problem management, and in some cases, these were unsolvable problems and unsuitable for the PM+ techniques. This finding supports the need for more focus on how to include financial problems in problem management in PM+. Greater training on how PM+ providers can select appropriate problems or how to address financial problems through the problem-solving strategy might serve to overcome this challenge. This may also reduce attrition rates as some participants might become frustrated by the lack of progress with key issues they felt negatively impacted their mental health, and could also alleviate CHV stress by the problems participants are facing.

Several limitations should be borne in mind when interpreting the results of this evaluation. Key-informant interviews are susceptible to bias, for example by informants giving answers that they thought the researcher wanted to hear, or not sharing certain information due to shame. We sought to minimize these biases somewhat by having an interviewer independent from the project conduct interviews. Furthermore, the translation of interviews from local languages to English may have lost nuances expressed in the original language. Another limitation is that only one rater, with limited experiences in the Kenyan context, conducted data analysis. Furthermore, due to the small number of key informants interviewed per stakeholder group, it is difficult to generalize results. Finally, not including the views of participants from the control condition, PM+ participants who dropped out of the study and those that declined participation in the study at the screening phase may have led to a bias in the results.

In sum, this evaluation provided insight into the factors perceived to be important when implementing multi-component interventions such as PM+. This data positively contributes to informing the successful integration of interventions such as PM+ and optimizing their impact in reducing the presence and interference of common mental health problems among people affected by adversity. The results also contribute to the evidence available to organizations and policy-makers developing services or integrating psychological interventions into LMIC health systems. It is recommended that future studies conduct comprehensive evaluations of implementation and integration of interventions into routine health care and identify the mechanisms and barriers to successful scale-up. An educational and possibly an incentive system may need to be built around the CHVs to deliver PM+ routinely effectively. On a policy level, it is recommended to give mental health more priority and to have the implementation of psychological interventions included in the mental health plan to allocate resources for implementation. Recognizing CHVs or similar cadres as part of the formal PHC system and putting in place a sustainable financing system will make the implementation of PM+ or similar interventions by non-specialist health providers.

## Supplementary material

For supplementary material accompanying this paper visit http://dx.doi.org/10.1017/gmh.2017.26. click here to view supplementary material
