# Supplementary material for: A qualitative evaluation of a brief multicomponent intervention provided by lay health workers for women affected by adversity in urban Kenya
Source: Glob Ment Health (Camb). 2018 Feb 6;5:e6. doi: 10.1017/gmh.2017.26 (PMC5827418; doi:10.1017/gmh.2017.26)
Supplement: Supplementary file 1 [file S2054425117000267sup001.docx]

**Annex 1: Qualitative evaluation**

| **Category of respondent** | **Topics to be explored** |
| --- | --- |
| CHWs | - Overall impressions of PM+ - Rapport with participants - Intervention adherence - Implementation of skills by participants |
| Intervention participants | - Overall impressions of PM+ - Rapport with facilitators - Intervention adherence - Burden of research interviews |
| Senior staff with policy implementation roles / connected to the research | - Existing scope of work of primary healthcare facilities:   *Considering existing primary healthcare clinics’ scope of work, do you view this additional intervention as something that can be delivered within the primary healthcare structures?*   - Integrating lay counselors into the PHC structures:   *How do you view the role of lay counselors (known as helpers in PM+) within primary healthcare?*   - Integration of PM+: - *What do you think of the PM+ programme in terms of ensuring high coverage of psychological problems?* - *Into what other programs (other than PHC) can PM+ be integrated?* |
